# Energy-Efficient Neuromorphic Architectures for Nuclear Radiation Detection Applications

**DOI:** 10.3390/s24072144

**Published:** 2024-03-27

**Authors:** Jorge I. Canales-Verdial, Jamison R. Wagner, Landon A. Schmucker, Mark Wetzel, Philippe Proctor, Merlin Carson, Jian Meng, Nathan J. Withers, Charles Thomas Harris, John J. Nogan, Denise B. Webb, Adam A. Hecht, Christof Teuscher, Marek Osiński, Payman Zarkesh-Ha

**Affiliations:** 1 Department of Electrical & Computer Engineering, University of New Mexico, Albuquerque, NM 87131, USA; jicv@unm.edu (J.I.C.-V.); jamisonrwagner@gmail.com (J.R.W.); lschmucker@unm.edu (L.A.S.); nwithers@unm.edu (N.J.W.); osinski@chtm.unm.edu (M.O.); 2 Department of Nuclear Engineering, University of New Mexico, Albuquerque, NM 87131, USA; mwetzel@unm.edu (M.W.); hecht@unm.edu (A.A.H.); 3 Department of Engineering & Computer Science, Portland State University, Portland, OR 97201, USAmerlincarson@gmail.com (M.C.); jmeng15@asu.edu (J.M.); teuscher@pdx.edu (C.T.); 4 Center for Integrated Nanotechnologies, Albuquerque, NM 87123, USA; ctharri@sandia.gov (C.T.H.); jnogan@sandia.gov (J.J.N.); dbwebb@sandia.gov (D.B.W.)

**Keywords:** neuromorphic computing, memristor arrays, radionuclide detection, radioisotope classification, source localization

## Abstract

A comprehensive analysis and simulation of two memristor-based neuromorphic architectures for nuclear radiation detection is presented. Both scalable architectures retrofit a locally competitive algorithm to solve overcomplete sparse approximation problems by harnessing memristor crossbar execution of vector–matrix multiplications. The proposed systems demonstrate excellent accuracy and throughput while consuming minimal energy for radionuclide detection. To ensure that the simulation results of our proposed hardware are realistic, the memristor parameters are chosen from our own fabricated memristor devices. Based on these results, we conclude that memristor-based computing is the preeminent technology for a radiation detection platform.

## 1. Introduction

Recent advances in neuromorphic computing (NC) facilitate the creation of massive brain-like parallel neural network (NN) computing systems. NC architectures, inspired by mammalian neuronal processes, can achieve classification tasks while consuming less energy than conventional computing systems [1,2]. In the past, NC has already achieved complex tasks like image and signal processing. Other applications include navigation, voice processing, and robot control. However, to the best of our knowledge, NC radionuclide detection has seldom been explored, despite the remarkable energy optimizations it would entail. A radiation detection sensor extracts the energy spectrum of the radionuclide radiation and compares it against a set of well-known radioactive materials. An application of a radiation detection sensor could be to measure and detect radiation in research equipment, such as X-ray diffraction (XRD) tools. Some radiation detection NN algorithms have emerged [3,4]. However, compelling NC architectures are yet to be designed. Based on our two proposed radionuclide identification NC architectures, we have determined that memristor-based computing is the preeminent choice for such a radiation detection hardware device.

Unconventional NC architectures could harness the memristor’s intrinsic computation dynamics to produce faster, cheaper, and more energy-efficient detection platforms [5,6]. Memristors have been applied for large random assembly networks [7,8]. Memristor-based systems with random or ordered networks have also executed simple pattern-classifying problems [9]. Moreover, NC system variation tolerance and parallel processing make memristor implementations suitable for radionuclide detection [10,11,12].

Our proposed memristor-based architecture aims to improve detection by providing single-chip bioinspired processing. The developed cross-point architectures allow for parallelism, thus increasing computing speed and bypassing the von Neuman bottleneck.

In addition to memristors, researchers have been demonstrating neuromorphic architectures with various types of nonvolatile memory (NVM) devices, such as floating gate transistors [13], organic field effect transistors [14], and memcapacitors [15]. However, the memristor’s real-time computing capabilities provide an advantage over other nonvolatile memories (NVMs) due to their low-power operation, which is the result of lower parasitic capacitance and smaller footprint [16]. Exploiting the memristor’s inherent device dynamics for intrinsic computation, certain tasks can be performed faster and more energy efficiently than with other NVM-based conventional architectures. Moreover, reservoir computing has lower learning complexity than traditional neural networks, because only the output layer must be modified or trained, as opposed to the entire reservoir structure. This is true independent of the types of devices used in the reservoir. In addition, memristor reconfigurability offers powerful self-healing properties that can protect against radiation-induced upsets or transients.

**Figure 1:**
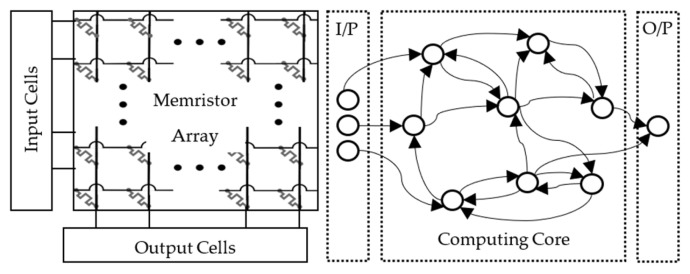
(**Left**) example device network; (**Right**) abstract computing setup using the device network on the left as a reservoir [17].

Figure 1 shows a high-level sample diagram for our network, with the memristor array as the reservoir “computing core”. This network is applied to identify radionuclide gamma-ray spectra. The system further establishes promising unconventional paradigms for NC architectures.

## 2. Materials and Methods

A wide range of applications implement NNs [18]. The NNs’ power is their ability to approximate arbitrary functions through a linear combination of weights and nonlinear activation functions. NC exploits NN characteristics through the use of vector–matrix multiplication (VMM) operations in the reservoir substrate. Our radiation detection system uses a single-layer NN inference application with rectified linear unit (ReLU) thresholding activation functions. Prior research by Li et al. implements multilayer neural networks with memristors [19]. Also, Bala et al. show simulation results of memristor approximation of the ReLU function [20]. It has been shown in [21] that a standard multilayer perceptron (MLP) network produces lower performance than the locally competitive algorithm (LCA). Therefore, our system’s NN is loosely based on Rozell’s LCA, an optimal solver for the sparse coding problem, which has been shown to be a promising approach in classification [22]. Mimicking the sparse neuronal activity in the mammalian primary visual cortex (V1), the LCA implements a sparse coding computation principle that has been harnessed to design our radiation detection system. The LCA implements local inhibition connections in neurons. These inhibitory interneural connections enhance the learning algorithm in NC hardware.

In LCA dynamics, an *m*th neuron’s receptive field, Φ*_m_*, contains a weight column vector that maps a particular dictionary class. In our system, each dictionary class corresponds to a specific radionuclide spectrum. The row elements in Φ*_m_* determine the sensitivity to each interacting presynaptic neuron. The input excitation signal vector proceeding from the presynaptic neurons is given by *s*(*t*), which contains sparse nonzero values. Each postsynaptic *m*th neuron’s excitation, ***b****_m_*(*t*), is given by <Φ*_m_*, *s*(*t*)>. Therefore, the strength of ***b****_m_*(*t*) is proportional to the similarity of *s*(*t*) to that neuron’s receptive field Φ*_m_*. Like in biological neural networks, each artificial neuron in our system charges up before firing. A time-varying internal state variable *u_m_*(*t*) contains the neuron’s accumulated charge. Then, a thresholding module monitors when the neuron’s ***u****_m_*(*t*) exceeds the threshold level *T_λ_* to activate that neuron and produce an output signal *a_m_*(*t*). The active neurons then compete among themselves through inhibition signals, which are proportional to both the activity level ***a***(*t*) and the receptive field similarity of competing neurons *G_m_*_,*n*_ = <Φ*_m_*, Φ*_n_*>. In summary, each neuron’s excitation dynamics is calculated by integrating a system of nonlinear ordinary differential equations [22]:

(1) $${\dot{u}}_{m}\left(t\right)=\frac{1}{\tau }\left[b_{m}\left(t\right)-u_{m}\left(t\right)-\sum_{m\neq n}G_{m,n}a_{n}\right]$$

The neuron(s) that best represent(s) the input signal will present fast-charging internal states ***u***(*t*), activating sooner and thus inhibiting the other slower-charging neurons. The inhibitory connections across all output neurons achieve sparse activity.

## 3. Results

Considering the potential benefits of using sparse coding algorithms to develop memristor-based reservoir computing systems, we present two architectures. While any device allowing in situ modifiable resistances would suffice to implement the NC reservoir substrate, we use the in-house fabricated memristor devices for the system’s assessment. Our analog and mixed-signal radionuclide identification architectures are described in Section 3.1 and Section 3.2, respectively. Details of the Al_2_O_3_/HfO_2_ memristor fabrication are given in Section 3.3. The characterization of the fabricated devices is discussed in Section 3.4. Finally, an expansion to radionuclide localization is presented in Section 3.5.

### 3.1. Analog Signal Architecture

**Figure 2:**
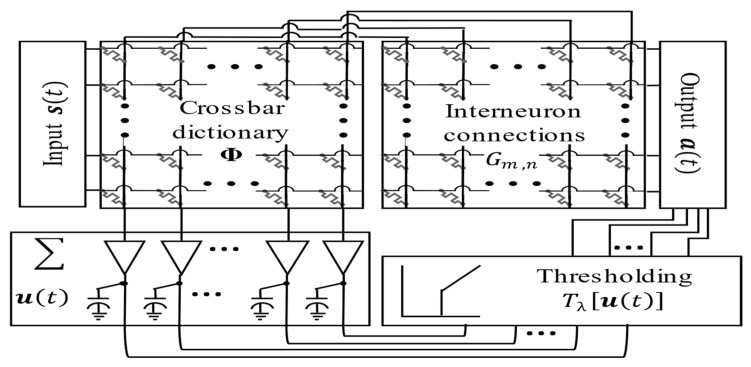
Block diagram of the LCA analog hardware circuit implementation [11]. The input *s*(*t*) is a vector containing all the detector channel analog voltage signals. The crossbar contains the neuronal receptive field weights Φ, mapped into each memristor state. The VMM determines the initial activation ***b***(*t*). An inverting amplifier operates as a virtual ground, which provides the sum (∑) of the current contributions from each memristor. The internal state capacitances are given by ***u***(*t*). A thresholding module containing a differential amplifier provides the circuit output ***a***(*t*), which is then fed back through the interneuron connections G to determine the inhibition signals.

Our first architecture described in [23] is expanded by implementing a memristor crossbar as the computing substrate. This system executes the LCA dynamics by harnessing the memristor’s long-term analog storage qualities. Figure 2 shows a simple block diagram model of all the necessary circuit components used.

All synaptic connections between sensory inputs and the processing neurons in this system are through the memristor crossbar dictionary Φ. The VMM operation in the memristor crossbar supplies each postsynaptic neuronal initial excitation ***b***(*t*). A correlation matrix containing weights proportional to the similarity of each receptive field pair *G_m_*_,*n*_ represents the inhibitory connections across all output neurons. Once the initial condition is set, the iteration in Equation (1) converges into a solution that resembles the best match to a set or combination of sets in the library.

The column header subcircuits accumulate and process the incoming currents to achieve a leaky integrate-and-fire (LIF) behavior. Each column header subcircuit contains an inverting amplifier for summation and scaling. Internal state capacitors model the algorithm’s time-varying internal state ***u***(*t*). A thresholding circuit *T*_λ_ obtains the final activation function ***a***(*t*). A second VMM between the correlation matrix *G_m_*_,*n*_ and the column header outputs ***a***(*t*) obtains the inhibition signal. The system offers a low-power solution to the sparse approximation problem. However, energy efficiency diminishes in larger systems due to the quadratic scaling of the number of circuit elements with the number of elements in the database N.

### 3.2. Mixed-Signal Spiking Architecture

**Figure 3:**
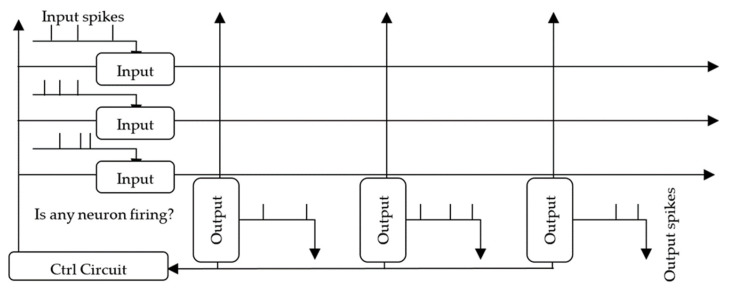
High-level diagram for our mixed-signal spiking architecture. During inference, input spikes pass through the presynaptic input neurons (Figure 4). Then, a signal is sent through the nanowire crossbar, where current signals pass through each memristor cross-point to charge and discharge the postsynaptic output neurons (Figure 5). When the output neurons fire, their spikes are propagated back through the crossbar into input cells as inhibition signals that are weighted by the memristor states. The control circuit monitors the output neurons to determine: “Is any neuron firing?”. When any of the output cells are firing, the input cells accumulate inhibition charge through feedback currents [17].

Figure 3 shows a high-level block diagram of this architecture suggested by Woods et al. [20]. To reduce power consumption and achieve the linear scalability of circuit elements with N, the system must calculate inhibition signals *G_a_*(*t*) without using additional interneural correlation *G_m_*_,*n*_ connection devices. Given that the analog signal architecture’s interneural inhibition connections *G* are a function of the dictionary crossbar Φ, we propose a system retrofit using simple spiking signals and a feedback path through Φ for inhibition signals. Without extra hardware, this mixed-signal spiking architecture uses a running time fraction to calculate inhibitory signals *G_a_*(*t*). Using the same memristor synaptic connections Φ for the forward system transmission, the scalability of the system becomes linear with N.

When one of the system postsynaptic neuron capacitors ***u***(*t*) charges above a certain threshold T_λ_, this neuron will activate the inhibition signal G*_m_*_,*na*_(*t*) feedback path through the same memristor receptive field crossbar-array column, Φ_firing_, it came from. The activation spike signals ***a***(*t*) pass current from the corresponding firing column, Φ_firing_, through the crossbar array in a backward path. The inhibition amount of each input signal depends on the firing neuron’s activation ***a***(*t*). This current will therefore charge the inhibition capacitors *C_inh_* in the input row, effectively blocking their signal from affecting the system. *C_inh_* blocks the presynaptic cell from transmitting the input signal *s*(*t*) row elements that are already accurately represented. The system then converges by suppressing the overrepresented signal row elements.

**Figure 4:**
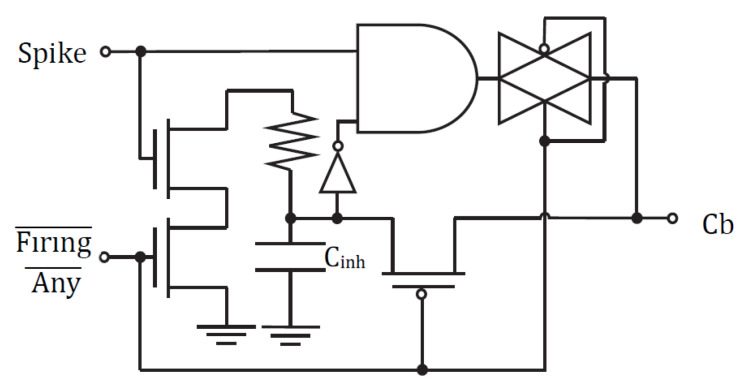
Row circuit input cell. This sensory neuron receives the rate-encoded spike signal and the control input $\bar{Firing\;Any}$, where the bar means negation. The neuron contains an inhibition capacitor Cinh, which charges when the cell’s signal is accurately represented in the system’s output. When any of the output neurons are firing, the control signal permits the charging of the inhibition capacitor through a feed backward path across the memristor array [17].

**Figure 5:**
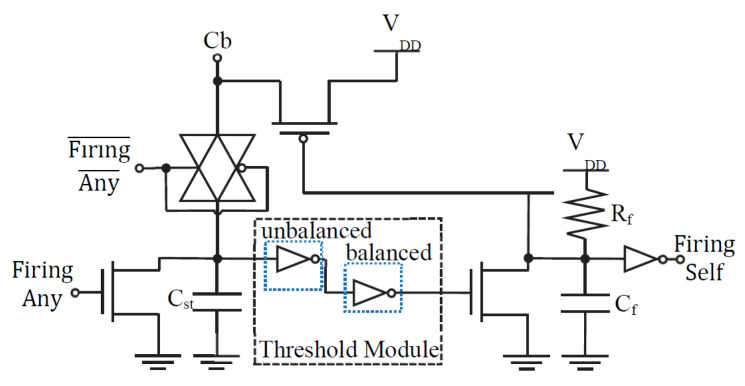
Column circuit output cell. Incoming charge from the crossbar input (*C*b) charges the accumulating internal state capacitor *C*_st_ through the transmission gate. When the capacitor’s charge surpasses a predetermined level, the operational thresholding circuit activates the cell and fires for a time interval determined by the firing resistor *R*_f_ and capacitor *C*_f_. The “Firing Self” outputs of all the system’s column neurons are sent to a NOR circuit (not shown) that determines the $\bar{Firing\;Any}$ control signal, which determines if the memristors are conducting in feedforward (i.e., row cells to column cells) or a feedback direction. The NOR circuit responds such that if there is any Firing Self signal, then it will be considered and feedback direction. Otherwise, it will be considered as feedforward [17].

Figure 4 and Figure 5 depict the basic cells that construct our mixed-signal spiking neuromorphic system. Figure 4 contains the row circuit for each of the system’s input cells. It is analogous to a sensory neuron in a biological system, where the rate of the spikes represents the strength (weight) of the triggering signal. Figure 5 shows the column postsynaptic neuron circuit, which involves the processing of logical neurons. Modeling the biological cell, the column circuit cell must charge itself before firing. In a biological NN neuron, the cell charges its body (or soma). In an artificial NN cell, a charging state capacitor ***u***(*t*) models this behavior. The neuron fires when its charge surpasses a certain threshold T_λ_. In a biological cell, the axon hillock executes the thresholding, whereas in our artificial cell, this operation is conducted by an unbalanced inverter.

The column circuit cell also contains a standard leaky integrate-and-fire (LIF) neuron setup, with the state capacitor connected through a transmission gate to the crossbar array. The transmission gate is analogous to the biological neuron’s somatic or dendritic cell membrane, which regulates the transmitters entering the cell. A Schmitt trigger setup allows enough firing time, such that the output state capacitor drains when the neuron fires, resetting all the accumulated potentials in the system neurons. Additionally, a pull-up transistor that sends the signal back to the crossbar array models the firing neuron’s axonal inhibition signal transmission. Therefore, during each neuron firing, an inhibition current flows back through the memristor into the row circuit cells. Since this charging occurs through a feedback path, the inhibition current flowing back into each row circuit is proportional to how well that row activated the firing column neuron.

The row circuit cell acts as the sensing neuron. It has a charging capacitor that models the cell’s body. However, in this case, the cell is actively transmitting a signal until it charges up with the inhibition signal blocking transmission. These row input cells discharge whenever an input spike signal arrives and charge when an inhibition output spike occurs. In other words, the inhibition capacitor charge models how much the output represents the output. This capacitor increases its inhibition voltage with the actual activity coming from the column cells through the crossbar junctions. The capacitor discharges through an inhibition resistor. Basic simulation results for these subblocks are provided in [24].

### 3.3. Al_2_O_3_/HfO_2_ ReRAM Crossbar Array Fabrication

**Figure 6:**
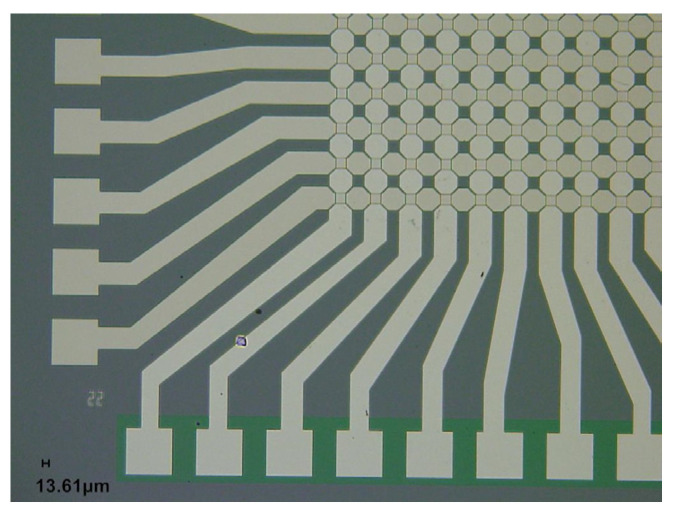
Optical microscope image of a completed memristor crossbar array showing the testing pads.

The fabrication of bilayer Al_2_O_3_/HfO_2_ memristor crossbar arrays was performed at the Center for Integrated Nanotechnologies (CINT), a user facility operated by the Sandia National Laboratories and the Los Alamos National Laboratory. First, we applied a hexamethyldisilazane (HMDS) coating and AZ 5214E photoresist to SiO_2_ over a Si substrate. Then, to define the bottom electrode patterns, we exposed the sample with 405 nm wavelength light for 6 s at 120 nJ using the Heidelberg Instruments 150 Advanced Maskless Aligner (MLA) optical lithography system. A Temescal FC-2000 metal evaporation system was used to deposit the Ti/Pt bottom electrodes. Excess metal and photoresist were removed using a liftoff process. Next, the samples were loaded into a Picosun SUNALE R150 atomic layer deposition (ALD) reactor. A 2 nm thick film of Al_2_O_3_ was formed through a stoichiometric process, using the chemical precursors trimethylaluminum and water at 250 °C. Subsequently, a HfO_2_ ultrathin film was formed using tetrakis (dimethylamido) hafnium (IV) and water at 250 °C, based on Molina et al. [25]. When the HfO_2_ film reached a 5 nm thickness, a blanket Ti/Pt layer was deposited using the metal evaporation system. The samples were then spin-coated with AZ 5214E photoresist and patterned with the MLA system a second time. Finally, the samples were mounted onto a carrier wafer, and the excess Ti/Pt metal was removed using an ion mill for 5 min. The crossbar arrays were wire-bonded to 44-pin LCC packages, the packages were sealed, and the arrays were electrically characterized. Figure 6 shows the microscope image of the fabricated memristor crossbar array, where the pads on the perimeter are used for electrical testing.

### 3.4. Characterization of Al_2_O_3_/HfO_2_ ReRAM Devices

**Figure 7:**
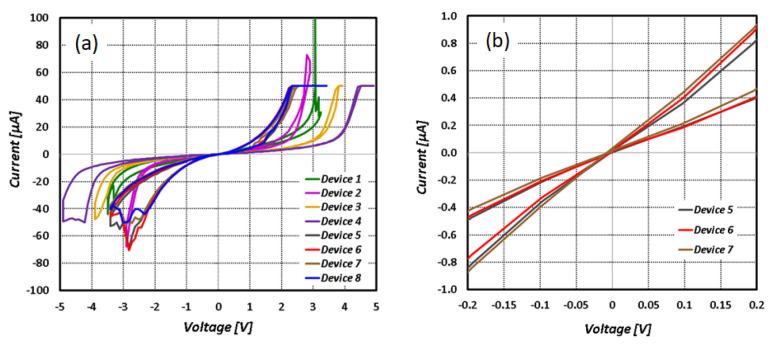
(**a**) multiple I-V hysteresis curves for 8 devices in a single column of an array of 11 μm × 11 μm memristors; (**b**) selected memristor devices (Row 1, Column 2) in the read mode.

Our fabricated memristors are bipolar, where the memristances are changed by changing the bias polarity. An HP 4145B Semiconductor Parameter Analyzer was used to characterize the memristor arrays. Figure 7 illustrates the results of iterative testing until device cycle reliability was found. To avoid possible dielectric breakdown, the compliance current was gradually increased from 10 µA to 50 µA. The steps to characterize the memristor were:

1. Form Step: The voltage was incremented in 0.25–0.5 V steps until a low-resistance pathway was formed, hitting a steady-state compliance current. We considered a device to be open when it failed to hit the compliance current despite the voltage being increased to 10 V.
2. Reset Step: Reverse bias voltage was applied using 0.5 V step decrements until the high-resistance state remained stable. The compliance current was disabled, as the current was throttled in a high-resistance state. However, a small risk of dielectric breakdown remained if the device state failed to change.
3. Cycle Step: A compliance current was set, and the on/off voltage was adjusted to the device switch values.

To avoid sneak path currents during characterization, the unselected crossbar rows and columns were electrically isolated by disconnecting relays on a custom-made testing board. Additionally, all cycled devices were left in the OFF state (i.e., the high-resistance state), such that the measured electric pathway corresponded solely to the tested device.

Figure 7 shows multiple I-V hysteresis curves for eight devices of a single column. The characterization results in Figure 7a show the full hysteresis curves of the memristor devices. All devices exhibited narrow hysteresis characteristics. The zoomed-in sections of the I-V curves in Figure 7b show the voltages at which the memristors can operate in the inference mode. For better clarity, only devices 5, 6, and 7 are shown in this figure. Notice that in the range from 50 mV to 100 mV, the memristors are usable for inference. The memristor fabrication yield for our samples was approximately 27% because several columns contained open-circuit line defects, likely due to metal trace discontinuities. In addition, the ratio of the high resistance (HR) to low resistance (LR) of our devices at room temperature was about 2×. Further refinement of the fabrication process will improve the overall yield and quality of the memristor devices. In an industrial setting, where the process variable is under better control using dedicated tools, the yield and device quality are expected to be significantly better than our lab setting using the same fabrication process.

The devices were not experimentally characterized under radiation. As with any electronics, we expect that intense gamma-ray radiation would create electron–hole pairs and may cause charging [26]. The current design considerations are not for the devices to be operated in a very intense field, such as in a very high contamination area or reactor core, in which there is no problem in finding the radioactive material. Rather, this is for low-energy, long-term remote detection and localization, which assumes lower-activity sources or material concealed with shielding.

### 3.5. Programming the Radionuclide Detection Dictionary

Programming the crossbar structure to represent radionuclide spectra tailors the system for detection tasks. We extracted 27 common radionuclides spectra, listed in Table 1, from the Nuclear Wallet Cards [27] and created a radioisotope dictionary. Our radionuclide-detecting system digitized the signals to 2048 energy bins. Therefore, each output neuron’s receptive field contained 2048 synaptic connections.

To determine the sparse weight matrix required for our application’s dictionary, we extracted the radioisotope event count number and distributed the hits uniformly across energy bins. Then, we fitted the counts to a kernel distribution and normalized (as a sum of squares) for identification speed.

The resulting dictionary contained 2048 × 27 weights mapped to a memristor crossbar. The memristor devices corresponding to energy bins containing major gamma-ray hits were programmed to the higher conductivity states. In contrast, memristor devices representing inactive spectral energy bins were programmed to lower conductivity states. Our detection system trained the conductance values offline by applying fixed training voltages into the crossbar through pull-up and pull-down transistors connected to each row and column.

### 3.6. Localization Task Algorithm

Localization algorithms use gamma-ray detector intensity and the inverse square law for operation. The radiation intensity is a function of the distance between the source and the detector. Our system sampled emission intensity values at a constant rate, while the detector moved along a linear path. This operation measured a trace of consecutive points in a specific region of interest.

The Intelligent Radiation Sensing System (IRSS) [28,29] dataset contains multiple experiments for configurations with different radionuclides, source activities, background profiles, and source/detector movement types. Our focus was on the Outdoor B14 Dataset, which used a 250 µCi Cs-137 source and 2″ × 2″ cylindrical sodium iodide (NaI) detectors. The B14 dataset consists of ten experiments with positions of eighteen detectors fixed and one source moving through the detectors at constant linear velocity. The only difference across the B14 experiments is the direction in which the source travels along the linear path and the fluctuations in measured gamma and background radiation.

**Figure 8:**
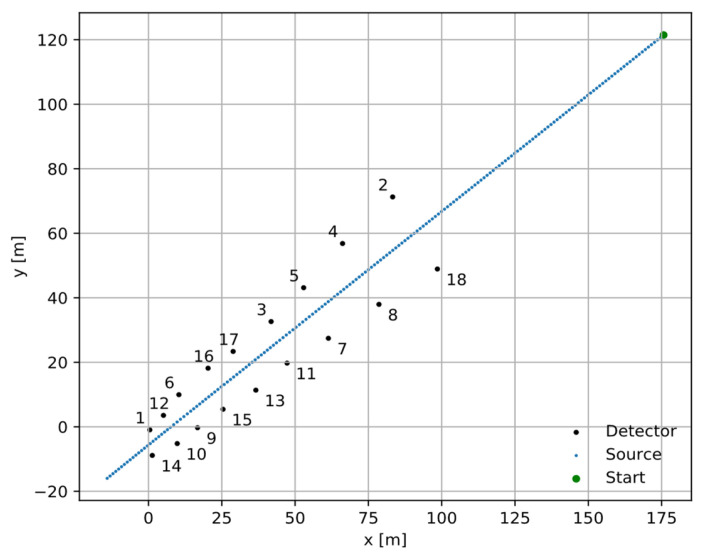
One of the ten experiments was conducted in the IRSS dataset with 18 detectors (shown as 1 to 18) and a single Cs-137 source moving with constant linear velocity. We switched the roles of the detector and the source for our analysis so that the detector moved along the linear path, and the source was fixed at the detector positions. This is a valid equivalence if the detectors are isotropic.

**Figure 9:**
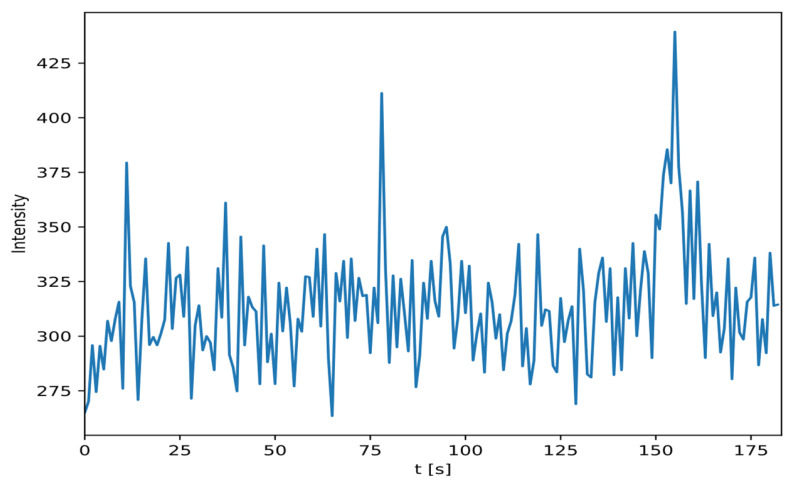
Measured intensity per timestep for position number 6 in Figure 8.

Figure 8 shows the original experimental setup, and Figure 9 shows a sample of the intensity measurement from detector position number 6 in Figure 8. We switched the role of the detector and source for our experiment so that the detector moved along the linear path, and the source was fixed at one of the detector positions per run. This reciprocal approach is valid because the detectors are isotropic to a first-order approximation, therefore the signal depends on the detector-source distance and not absolute position or orientation. These result in 18 unique runs for each of the 10 experiments for a total of 180 runs. For each run, the number of measurements was reduced to 60 samples, where the detector passed by the source to fit our proposed network architecture size.

A comparison baseline was established with a Markov Chain Monte Carlo (MCMC) algorithm known as Adaptive Metropolis sampling [30]. The MCMC model is given the source activity and background rate, so that it just needs to estimate the source location, effectively serving as an “oracle” model. This gives a practical lower bound on the achievable performance of any model and aids in demonstrating the system’s performance.

## 4. Discussion

### 4.1. Radionuclide Classification

**Figure 10:**
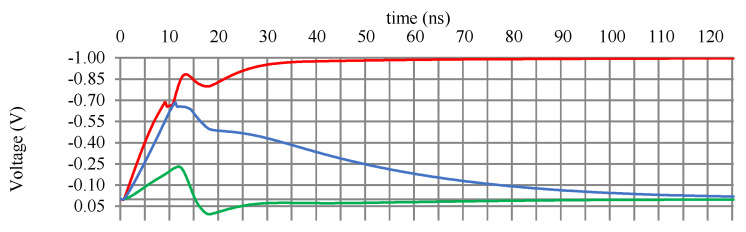
SPICE simulation result for the analog architecture. The two representative outputs (2 and 5), shown in red, successfully converged into −1 within 30 ns. The other outputs, shown in green and blue, converged into 0. The output was negative due to the inverting amplifiers in the column cells.

**Figure 11:**
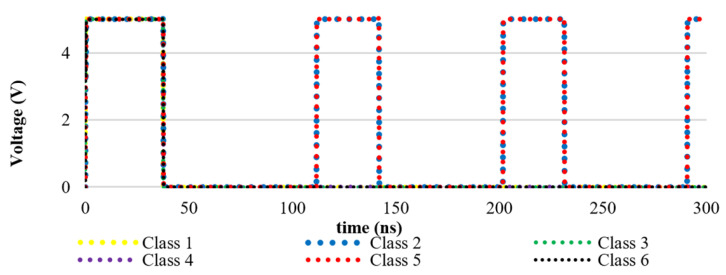
SPICE simulation of the spiking architecture. The two representative outputs (2 and 5) were detected. The other outputs (1, 3, 4, and 6) were inhibited.

As a proof-of-concept demonstration of the NC architecture, we simulated a simple system using SPICE. To develop our simulation blocks, we implemented the architectures described in Section 3.1. For circuit-level simulation within a reasonable computation time, a simple LCA system containing six neurons with five-element receptive fields (RFs) was constructed, where the implemented dictionary was a mathematical representation of six classes, including 10000, 01000, 00100, 00010, 00001, and 11111, numbered 1 through 6, respectively. It is expected that, e.g., if 01001 is given as input, the LCA should identify both classes 2 and 5 as the likely input. We assessed both architectures with this task. In both architectures, the circuit successfully identified both classes. Figure 10 and Figure 11 show the time response diagrams of each system.

Note that although the SPICE simulations presented in this section were performed on an ideal LCA system, memristors are typically faulty and defective. However, due to the massive parallelism, the NC architectures are typically resilient to faults and defects. A more comprehensive simulation and analysis is demonstrated in [11], where the impact of nonideal memristors and their defects in a neuromorphic radionuclide identification system is presented.

Figure 10 and Figure 11 show the SPICE simulation results for the analog neuromorphic architecture. The two representative outputs (2 and 5), shown in red, successfully converged into −1 within 30 ns. The other outputs, shown in green and blue, converged into 0.

### 4.2. Radionuclide Localization

**Figure 12:**
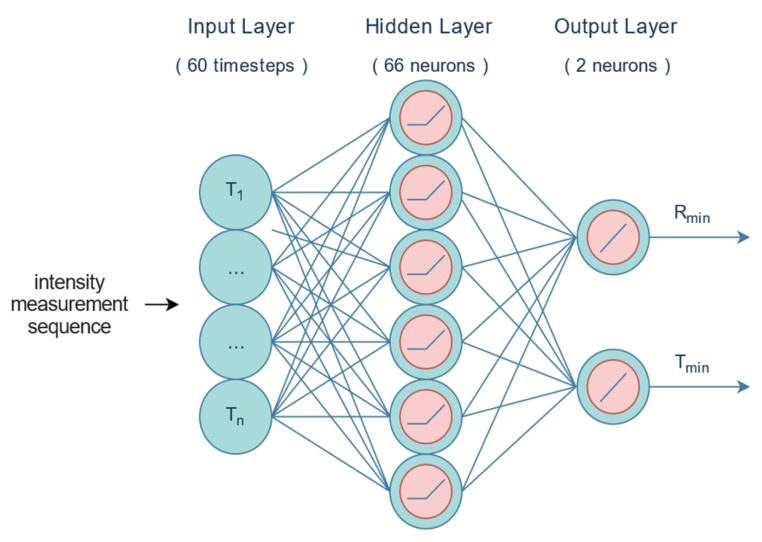
NN architecture for localization algorithm. The NN input is 60 samples, at a fixed time interval of a radionuclide’s intensity along a gamma-ray detector’s trajectory. The intensity levels are passed to all 66 neurons in the first hidden layer. All neurons in the hidden layer are fully connected to the two output neurons. The output of the NN is the highest intensity level timestep (*T*_min_), and the radionuclide emission source radius is recorded (*R*_min_). All neuron signals in the hidden layer are passed through a ReLU activation, while the output neurons are linear.

**Figure 13:**
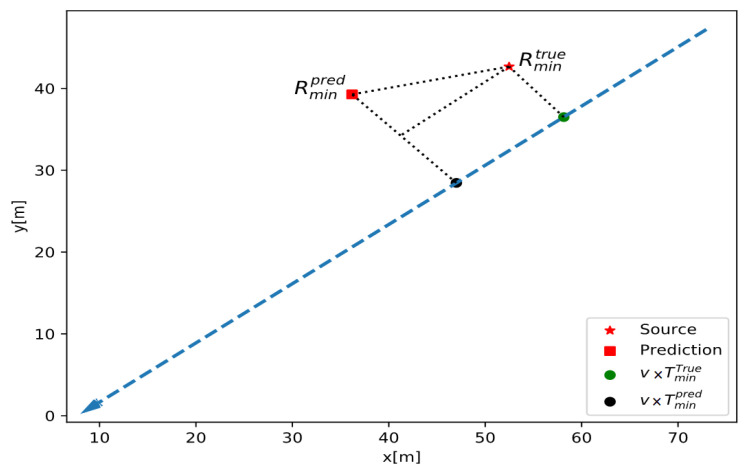
The prediction quantity $R_{min}^{pred}$ is the predicted minimum distance between the source and detector line of motion during a measurement sequence, and $T_{min}^{pred}$ is the predicted time at the closest approach. The quantities are converted to a total difference distance, the prediction error. The time and minimum distance are treated as independent and orthogonal; hence, the prediction error is given by Equation (2).

Figure 12 shows a fully connected NN with 60 inputs and 2 outputs trained in software to perform the localization tasks. The model is trained on the simulated data of a detector moving linearly with constant velocity past a radiation source. The hidden layer consists of 66 neurons. The signal at this hidden layer is a linear combination of 60 intensity measurements, learned weights, and biases, which is then passed through the ReLU activation function (rectifier), which performs the operation *f*(*x*) = max(0,*x*). These signals are then multiplied by their corresponding neuronal output layer weights and summed to attain *T*_min_ and *R*_min_. The source location is predicted with T_min_ and R_min_ because the linear path of the detector does not allow disambiguation of which side of the detector the source is on. Thus, *R*_min_ is the minimum distance between the source and the detector during a measurement sequence, and *T*_min_ is the corresponding time when this occurs (see Figure 13).

*R* and *T* values are independent in this architecture. The prediction quantities are converted to a difference distance, called the prediction error, and given by the following equation [31]:

(2) $$Dist\;=\;\sqrt{{\left(R_{min}^{True}\;-{\;R}_{min}\right)}^{2}+{\left[{\left\|{(T}_{min}^{True}-T_{min})\times v_{detector}\right\|}_{2}\right]}^{2}}$$

where *v_detector_* is the constant detector velocity, and ||.||_2_ is the Euclidean norm. The performance results using the IRSS Outdoor B14 test set are summarized in Table 2. The “Closest Distance” column denotes the distance of the closest approach between the detector and the source for a run. For example, of the 180 total runs across the 10 experiments, 44 had the detector come within 3–5 m of the source position.

The left column shows our binning criterion, i.e., the closest distance between the detector and the source for a run and the number of runs in the bin. The middle and right columns give the total difference distances for the MCMC and NN simulations.

### 4.3. CMOS Neuron Energy Consumption per Spike

Several new and novel neuromorphic devices have recently been developed, such as conventional planar organic field-effect transistors (OFETs) [14] and multisensory neuro-morphic devices [32] that allow for ultralow energy consumption. However, due to the compatibility with the mainstream microelectronic production, CMOS neurons are still more desirable.

We optimized the energy and area efficiency of the mixed-signal CMOS neurons shown in Figure 4 and Figure 5. Specifically, these CMOS integrate-and-fire neurons have a minimum number of transistors. The main energy consumption of the neurons occurs due to the charge/discharge of the capacitors. Hence, we aimed to minimize the capacitor dimensions to optimize the energy efficiency of the neurons. Our spiking mixed-signal neurons have accumulation, firing, and idle modes of operation.

Accumulation occurs when the neuron receives and integrates the charge carried by a spike signal through the memristor synaptic weight. The postsynaptic column neuron operates primarily in accumulation when a signal is sent by the presynaptic neuron through the crossbar. Presynaptic row neurons only operate in accumulation when receiving inhibition signal feedback through the memristor crossbar.

Firing occurs when the neuron produces a spike. The generation of the postsynaptic neuron spike determines the energy consumption through the output spike characteristics. The presynaptic row cell operates primarily in the firing mode, transmitting spike signals when the row is not inhibited. The postsynaptic column cell fires only when the neuron has accumulated charge above the threshold level, generating an inhibition spike signal output that is feedback through the crossbar.

The idle energy is dominated by CMOS leakage. The CMOS neuron does not consume energy during learning, unlike the memristor synapse. Table 3 shows the per-spike energy consumption of the input row and output column neurons.

### 4.4. Overall System Energy Consumption

Mapping a memristor crossbar array is straightforward and efficient. Each linkage between neuron layers requires two memristors, along with an additional one for the layer’s bias value. The measured radiation intensity determines the signal s. The memristor states contain the learned positive (Φ+) and negative (Φ−) weights. *b*− and *b*+ are given by the dot product between the radiation signal *s*(*t*) and the memristor weight crossbar columns, i.e., *b* − (*t*) = <Φ−, *s*(*t*)> and *b* + (*t*) = <Φ+, *s*(*t*)>. The ReLU activation function is obtained using a memristor ratioed logic MIN activation function [33] and a comparator, as proposed in [16]. This circuit yields f(*x*) = max(0,*x*). The comparator has *b*− and *b*+ as the inverting and noninverting inputs, respectively. The MIN circuit uses two memristors connected to the neuron’s output node. Memristor M1′s input is the comparator output, while M2’s input is *b*+. If *b*+ is greater than *b*−, then *b*+ is the output; otherwise, it is zero.

Mapping our NN to hardware requires a memristor crossbar array containing 8186 memristors, with 61 × 132 at the hidden layer and 67 × 2 at the output layer. This structure fits on a single 128 × 64 array, similar to the one used by Li et al. [19]. According to Chakma et al. [34], the typical per-spike energy usage for an active Al_2_O_3_/HfO_2_ memristor synapse during inference is approximately 0.48 pJ. Assuming all synapses are active during an inference operation, we estimated a network energy cost of approximately 3.9 nJ per operation.

## 5. Conclusions

Using Al_2_O_3_/HfO_2_ memristor arrays, we designed and simulated a neuromorphic system for radionuclide detection. The analysis demonstrates that the classification and localization tasks are achievable with minimal energy consumption using the developed neuromorphic architectures. Moreover, considering that the device will be fabricated on a standard CMOS process with a CMOS-compatible memristor fabrication, we anticipate that the sensor cost will be minimal. Through the testing of our two proposed radionuclide identification NC architectures, we have determined that memristor-based computing is an energy saving choice for radiation detection hardware.

## Figures and Tables

**Table 1:** Well-known radionuclides [11,27].

Nuclide	Major γ-rays (keV)	Nuclide	Major γ-rays (keV)
Na-22	511, 1275	Cr-51	320
Mn-56	847, 1811, 2113	Fe-59	1099, 1292
Co-57	122, 136	Co-60	1332, 1173
Cu-64	511	Ga-66	511, 1039, 2752
Ga-67	93, 185, 300	Ga-68	511, 1077
Se-75	265, 136, 280	Sr-85	514
Ru-103	497, 610	In-111	245, 171
I-123	159	I-131	364, 637, 284
Cs-137	662	Ba-133	356, 81, 303
Ce-144	134	Sm-153	103, 70
Eu-152	122, 344, 1408	Ho-166	81, 56
Yb-169	51, 63, 57	Ir-192	317, 468, 308
Tl-201	71, 69, 80	Bi-207	570, 1064, 75
Am-241	60		

Major γ-rays are ordered according to intensity.

**Table 2:** Localization simulation results on the IRSS Outdoor B14 Test Dataset [29].

Closest Distance [m]	MCMC [m]	NN [m]
3–5 (44 runs)	2.41	3.02
5–7 (37 runs)	3.66	4.48
7–9 (44 runs)	5.35	5.75
9–11 (22 runs)	7.80	7.58
>11 (33 runs)	11.05	9.73
Average	5.63	5.78

**Table 3:** Per-spike energy consumption values for CMOS neurons.

Presynaptic Neuron		Postsynaptic Neuron	
Neuron Phase	Energy per Spike (pJ)	Neuron Phase	Energy per Spike (pJ)
Accumulation	1.1	Accumulation	21.8
Idle	negligible	Idle	3.5
Firing	17.2	Firing	140.6

## Data Availability

Some data was also extracted from the NNDC website. Radionuclide Spectral Data is available here: as https://www.nndc.bnl.gov/. The Localization Experiment Data Sets are available here: https://github.com/raonsv/canonical-datasets.
